# A Rhumba of “R’s”: Replication, Reproducibility, Rigor, Robustness: What Does a Failure to Replicate Mean?

**DOI:** 10.1523/ENEURO.0072-16.2016

**Published:** 2016-07-07

**Authors:** Oswald Steward

**Affiliations:** Departments of Anatomy and Neurobiology, Neurobiology and Behavior, Neurosurgery and Center for the Neurobiology of Learning and Memory, Reeve-Irvine Research Center, University of California, Irvine, California 92697

## Significance Statement

Widespread reports of failures to replicate have undermined confidence in published scientific findings. This is especially critical for preclinical studies that were seen as steppingstones to novel therapies. Recognizing the importance of publishing replication studies and reporting negative data, *eNeuro* will consider such articles; failed preclinical tests are especially welcomed. Here, I consider how failures to replicate should be interpreted and suggest possible new journal practices.

## Introduction

“Rhumba” is a collection term for a group of rattlesnakes, and there is growing concern about a rhumba of “R’s” (replication, reproducibility, rigor, robustness), AKA, the “replication/reproducibility crisis”. Widespread reports of failures to replicate key findings have undermined public confidence in scientific research. Concerns about lack of reproducibility have led to initiatives by scientific societies, journals, and funding agencies to improve scientific rigor with the assumption that this will improve reproducibility.

One of the explicit editorial policies of *eNeuro* is to consider articles reporting negative results and failures to replicate; failed preclinical tests are especially welcomed. Given this, it’s important to consider how one should interpret failures to replicate when they are published.

## Interpreting failures to replicate

The first thing to emphasize is that a failure to replicate doesn’t mean that there is any suspicion of scientific misconduct. A failure to replicate a study published in a peer-reviewed journal simply means that similar results were not found. There are many reasons why this may be the case including that the initial study is correct and the replication study is flawed. Thus, a failure to replicate is simply a call to attention that there is a discrepancy.

When fundamental biological findings are not replicated, science will hopefully self-correct eventually (but see, Ioannidis, 2012). In the interim, faulty conclusions impede the advancement of knowledge and may lead to further faulty conclusions. In studies involving animal models of diseases or disorders, promising findings can lead to attempts to translate to therapies, which can waste a lot of money on dead ends if the original findings are impossible to replicate. Wasting money on preclinical studies of therapeutic candidates is one thing, but launching early stage clinical trials on ineffective therapies is another thing entirely. Although not always fully understood, the primary concern of regulatory agencies like the Food and Drug Administration (FDA) is safety, not efficacy. If the scientific basis of a therapeutic candidate isn’t strong, lack of efficacy may not be discovered until phase II or beyond, by which time a lot of money has been spent, and subject participation has been wasted.

The fact is that some clinical trials have been launched based on fundamental research that has not been replicated in independent published studies (key words here being “independent” and “published”). Independent replication is not a prerequisite for FDA approval of a clinical trial, and research performed by companies that support the development of new therapeutics may not be published in peer-reviewed journals at all, much less independently replicated. There is speculation that one reason for the high failure rate of clinical trials is that the scientific data that underlie the trial were weak in the first place. This explains the growing belief that replication of foundational experiments and reporting negative data are important.

In considering failures to replicate, one first step is to check the original report and the replication for “Begley’s 6 red flags” (Begley, 2013): (1) Were experiments performed blinded? (2) Were basic experiments repeated? (3) Were all the results presented? (4) Were there positive and negative controls? (5) Were reagents validated? (6) Were statistical tests appropriate? A positive change in publishing practice would be to check submitted papers for these red flags, and if present, require consideration of resulting caveats in Discussion sections.

The presence of red flags doesn't mean a paper is invalid. As discussed previously with examples (Steward et al., 2012), it not always possible to be blind especially when effect size is large. Red flags do mean that caveats should be noted in Discussion sections.

A second step is to consider the statistical power for both original and replication studies. Scientists worship statistical significance, assessed by *p* value, but findings that are statistically significant are not necessarily well powered. It is often wrongly assumed that statistical power is only related to the risk of failing to detect a difference when one is present. In fact, low power also increases the chance that a statistically significant finding is actually false. One meta-analysis of 730 studies in neuroscience revealed a surprisingly low statistical power (median power of 21%; Button et al., 2013). If this is representative, it is not surprising that some findings are not replicated. A second positive change in publishing practice would be to require that papers report statistical power, as well as *p* values.

This raises another point; the misuse and misinterpretation of *p* values. Concerns about this issue led to a position paper published on behalf of the American Statistical Association (ASA) on *p* values and statistical significance (Wasserstein and Lazar, 2016). Concerns about over-reliance on significance and *p* values have led some journals to explicitly prohibit reporting of *p* values. Without getting into the weeds of statistics, there are a few important points: (1) a smaller *p* value does not mean that differences are greater; it means that there is a higher statistical probability that there actually is a difference between groups. Stated another way, *p* value is not a measure of the magnitude of differences. (2) If *p* > 0.05, it does not mean that groups are the same. Lack of statistical power can lead to failure to detect an actual difference between groups (a type II statistical error). As the ASA position paper notes: “A conclusion does not immediately become ‘true’ on one side of the divide and ‘false’ on the other.”

It is worth considering whether there have been pressures that impacted on statistical power of studies in recent years and reduced self-replication (Begley’s red flag #2). One pressure comes from another “R” to add to the rhumba. This is from the guiding principles on the use of animals in research (reduce numbers of animals). Committees that review animal research protocols (institutional animal care and use committees or IACUCs in the United States) require steps to reduce numbers, and avoid “duplication”. Of course, repeating experiments *is* duplication. Addressing the “R’s” in guiding principles for animal research may have had the unintended consequence of reducing power and self-replication.

Next, it’s important to drill down into how the original and replication experiments were actually done. One important question is whether the original study was a “rolling experiment”. Many papers, especially on “novel findings”, report results from experiments where a few animals/analyses are done here, a few there, data and groups are compiled as you go, key control groups are added as you realize you need them, there are interim statistical analyses, and subjects/analyses are added until differences reach statistical significance (testing to a foregone conclusion). This is almost never reported in Methods; instead, Methods sections read as if the study was preplanned and all groups were run simultaneously. So, an important comparison between original and replication is timing of data collection and compilation of groups, particularly whether critical experimental and control groups and analyses were run at the same time.

If groups are compiled over time, it’s important to know whether each run of the experiment included subjects from each group or whether different groups were done at different times. Because Methods sections in many journals have become abbreviated and are often relegated to supplemental information, it may be impossible to find out about timing unless one asks the authors directly. Hence, the third positive change in publishing practice would be to require information on timing and group compilation in the Methods, or at least note that data were collected over time, and provide details on timing in the supplementary data. In these days of electronic publishing, omitting methods is by choice, not economic constraints.

One other question that often can’t be answered without asking the authors directly is whether all analyses were reported. The issue here is only presenting the data that show statistically significant differences and not other assessments that were done. Multiple assessments increase the risk of false-positives due to type I statistical errors (a significant difference between groups that is actually due to chance). As a specific example, studies of locomotor recovery following spinal cord injury may assess many aspects of hindlimb locomotion (BBB score, sub-score, stride length, stride width, toe spread, speed, and more). Of course, recovery on any variable could be important, but if you measure 20 things, then it is expected that one of the 20 observations (0.05) will be different by chance. Thus, a *p* < 0.05 difference on 1 measure of 20 is highly likely to be a type I statistical error. This emphasizes the importance of disclosing all analyses that were conducted (Begley’s red flag #3), which is one aspect of transparent reporting (Landis et al., 2012; Wasserstein and Lazar, 2016 provide further discussion of the problem of multiple analyses).

Of note, when there are multiple analyses, techniques used for analyses of big datasets (determination of false discovery rate) could be useful. Depositing raw data online would provide an opportunity for reanalysis of data in different ways by others, which could help resolve any discrepancies between an original and follow-up study.

Equipped with as much detailed information about methods as possible, critical readers can then make judgments about how similar the original and replication studies actually were.

Responses to reports of non-replication often are “You didn’t do it exactly the same way”. Fair enough; it’s impossible to do studies in exactly the same way in two different laboratories. Scientific methods are complicated and the “replicating” laboratory may not be as experienced in the techniques as the laboratory that published the original study. There are likely to be subtle but important differences in laboratory procedures even when detailed SOPs are written out. Also, practices evolve over time, often without being recognized by the individual and certainly off the radar screen of a senior principle investigator. For difficult procedures requiring a high degree of skilled motor function (animal surgery for example), there is a learning curve. For experiments done over time, there is evolution of skill and may be personnel change. Even more difficult to control is the fact that skilled performance may evolve over the course of a single day as the individual “gets into the groove” or becomes fatigued, so another variable is the number of procedures that are done on a single day.

For studies involving live animals, uncontrolled variables include housing conditions (caging and number of animals per cage), handling by vivarium staff, where and when experiments are done (time of day, features of testing area), procedural differences in functional (behavioral) testing (Sorge et al., 2014), qualitative judgments in rating scales, phase of the moon (really), and even the clothes that laboratory staff wear. A recent report indicates that results in studies of pain depend on the gender of the laboratory staff, and are different even when a woman wears clothing previously worn by a man. Similar issues apply to cells growing in culture. Cell culturing is an art, and reliable cell cultures require skills learned by extensive practice. Practices evolve over time, sometimes in ways that aren’t noticed. Changes in journal policy could advance understanding of key biological variables by requiring full methodological details, including describing actual protocols rather than referring to previous published articles and noting other details discussed above.

But there’s an important conclusion to be drawn, and this is where another “R” comes in; *robustness*. If results depend on the exact conditions of the experiment, then the results are not robust; they’re “conditional”. The conditions may be very narrow or fairly broad, but if the original findings were interpreted as demonstrating general principals, then broad-reaching claims probably need to be revised.

Broad claims are sometimes explicit and sometimes implied in studies of interventions in animal models of disease. Often, such studies are interpreted as pointing the way toward novel therapies. However, if findings are “conditional” then the discovery is probably not relevant for translation. The more narrow the conditions need to be to demonstrate an effect in an animal model, the less likely it is that the manipulation is a reasonable therapeutic candidate. Manipulations that only work in constrained circumstances are almost certain to fail in the highly heterogeneous setting of human disorders.

If there is an explicit or implied claim of broad relevance, is it reasonable to respond to a failure to replicate by saying that “you didn’t do the experiment the same way” (meaning that the findings are conditional and apply only in a particular set of experimental conditions)? This highlights another trend; the missing “caveats” section in Discussions. For any novel finding based on a limited dataset, it would be more prudent to say that the findings *might* only apply in highly constrained circumstances until proven otherwise.

If effects depend on the exact conditions of an experiment, then the next step to advance understanding is to define the critical conditions. In this way, a failure to replicate is the first step in the iterative advancement of understanding of the underlying biology; failed replications help define the limits and parameters of the biological phenomena.

In an ideal world, a failure to replicate should be followed-up by studies to define the conditions in which a finding applies versus conditions in which it does not. This was not possible in the NIH replication contracts because funding was limited to the replication itself; funds could not be used for follow-up experiments to explore the biology. This raises the question of who should do such follow-up studies and who should pay for them. If the original finding is of key importance to the initiating laboratory, then it seems reasonable that the ball is in their court.

This brings us to an “F” rather than an “R” word (funding). How will these studies to resolve discrepancies be funded? Funding agencies focus on innovation and novelty but we can't move the field forward by focusing exclusively on novelty. Enduring advances are made by resolving discrepancies.

### Perverse incentives

Perverse incentives have been discussed primarily in terms of rushing things to meet deadlines for job applications, grants, promotion, and tenure consideration, etc. Journals can’t change these, but one thing journals can change is “experiment by reviewer”. Most of us have given and received comments like: “To make the case stronger, the authors need to show XX”. Now there’s a perverse incentive; get these results and your paper will be accepted! Unbiased experimentation requires that any outcome be acceptable. Reviewers’ practices probably can’t be changed, but a positive change in journal policy would be to require that “experiments by reviewer” be noted as such.

Scientific rigor can certainly be improved but it remains to be seen whether enhancing rigor will actually enhance reproducibility. Perhaps our novel discoveries are about biological phenomena that are so complex that most findings will be conditional (that is, depend on circumscribed conditions). If so, enhancing rigor may improve the quality of published papers without making the findings more reproducible. Time will tell.

Some suggested changes in publishing practices:

1) Check submissions for “Begley’s 6 red flags”, and if present, require consideration of resulting caveats in Discussion sections.
2) Require that papers report statistical power.
3) Require statements about whether studies were done as “rolling experiments” and require information on timing of data collection.
4) Require that all analyses be reported.
5) Require a caveats/scientific rigor section in Discussions.
6) Require specific indication of studies performed at the request of reviewers.

In contradistinction to the six red flags, the points above could be called “the 6 gold stars for rigor”.
